# Bite-size research: *BMC Research Notes* goes back to its roots

**DOI:** 10.1186/s13104-017-2418-y

**Published:** 2017-02-14

**Authors:** Dirk Krüger, Diana M. Marshall

**Affiliations:** BioMed Central, 236 Gray’s Inn Road, London, WC1X 8HB UK

## Abstract

Since it first launched in 2008, *BMC Research Notes* has been a place where researchers can publish short notes and observations, research outputs which are useful for the community but which can end up hidden in the lab notebook or as a footnote in a dataset. In order to re-affirm the importance of publishing these kinds of outputs, the journal is renewing its focus on publishing note articles as well as other potentially dark data such as short null results. Publishing these articles is useful for many researchers, therefore we are also expanding the scope to all scientific and clinical disciplines including health sciences, life sciences, physical sciences, mathematics and all engineering disciplines. With this refocusing of *BMC Research Notes* back to its original vision, BioMed Central is offering a home for short communications to make dark data and single observations widely available to the global research community.

## Background

*BMC Research Notes* was launched in 2008 as part of the *BMC* series to provide a home for short publications, case series, incremental updates to previous work, results of individual experiments, and similar material that lacked a suitable publication outlet [1]. The intention was to reduce the loss suffered by the research community when such results remain unpublished. In comparison to other *BMC*-series journals [2] which cater to specific research communities, *BMC Research Notes* has been considering submissions across all fields of biology and medicine. In 2011, as the journal had begun to receive full-length research articles, both as direct submissions and transfers from other journals, we broadened its remit to include such articles. Publishing between 500 and 1000 articles each year since 2011, *BMC Research Notes* has grown into a highly successful open access journal.

Whilst there has clearly been a need for *BMC Research Notes* to consider full-length research articles, doing so has masked the original vision of the journal as something different; a place where short observations, which might otherwise be unknown, can be made available to the global research community. Since the original launch of *BMC Research Notes* there has been an increased interest in journals which provide alternative publication options for authors, reflected in the launch of journals such as Research Ideas and Outcomes [3] and Matters [4]. There is also increased discussion of the need to provide suitable publication destinations for null results. The Journal of Negative Results in BioMedicine [5] has provided a destination for this since 2002 and more recently PLOS ONE launched its ‘Missing Pieces’ collection [6] to highlight this issue. In the light of these discussions, it is the right moment to address the focus of *BMC Research Notes* and bring it back to its original vision.

We are still committed to providing a home for all scientifically valid research outputs across the *BMC* series. Full-length research articles which would previously have been considered by *BMC Research Notes* can find a home in the relevant subject journals in the *BMC* series as outlined in a recent blog [7] discussing the editorial threshold for these journals.

### Freeing dark data

Within scholarly publishing, the phrase dark data has been used to describe research outputs that are not usually considered in traditional research journals such as single observations. As a result, these research outputs often never see the light of day and remain hidden in researchers’ lab books or buried as some extra data to be considered further one day in the future.

At the beginning of a new research project, researchers often try to replicate experiments previously reported in the literature to test their methodologies. Confirmatory or discrepant results are of immense value to the research community. Data can also be received as a ‘side product’ of a larger study. This so-called orphan data was not expected and researchers may be unsure how to interpret it. However, we believe that orphan data should be made available as other researchers, if not now then perhaps in the future, might have the tools and knowledge to gain valuable insights. Null or negative results are produced by all researchers in every research institution across the world. It is the bread and butter of research to try new ideas and more often than not these lead to a cul-de-sac that many journals will not consider. However, it is extremely valuable to report what does not work and to make that knowledge widely available so others can learn from it and advance our knowledge and understanding.

There are many advantages in helping researchers to publish their dark data such as the reduction in duplicating research and the dissemination of fragmented findings or null results potentially useful to the research community.

## The “new” *BMC Research Notes*

We are bringing the journal back to its roots with a focus on shorter research notes that aim to bring dark data out of the shadows. The journal will once again focus on less conventional research outputs such as updates to previous work, single observations and null results that are often overlooked.

We believe that there are strong incentives for researchers to make their dark data available by publishing in *BMC Research Notes*:

- Service to the research community by making valuable data available
- Turn data already collected and individual results already obtained into a publication
- Published articles could potentially attract a high number of citations.

Most research journals within the scholarly publishing landscape favor manuscripts that tell a narrative. Authors introduce the topic; provide an overview (selected literature review) of where the field currently stands and identify the knowledge gap (research question). The methodologies and results presented fill the knowledge gap and authors conclude with an outlook for further research. *BMC Research Notes* will publish articles without bias and we take the focus away from story-telling to making all valid research outputs freely and openly accessible.

### Aims and scope

*BMC Research Notes* publishes scientifically valid research outputs that cannot be considered as full research or methodology articles. We support the research community across all scientific and clinical disciplines by providing an open access forum for sharing data and useful information; this includes, but is not limited to, updates to previous work, additions to established methods, short publications, null results, research proposals and data management plans.

As the pioneering and original open access publisher, we believe that open access and the Creative Commons Attribution License [8] are essential in this, allowing universal and free access to all research notes published in the journal and allowing them to be read and the data re-used without restrictions.

The journal is overseen by a professional Editor with a scientific background along with an expanding international team of Associate Editors [9]. All manuscripts submitted to *BMC Research Notes* must comply with our editorial policies [10]. Particular emphasis is given to appropriate ethical approval and consent for manuscripts describing research involving humans (including human material and data), animals (including regulated invertebrates) and plants. All manuscripts will be peer reviewed by appropriate experts.

### New article type ‘Research note’

**Fig. 1:**
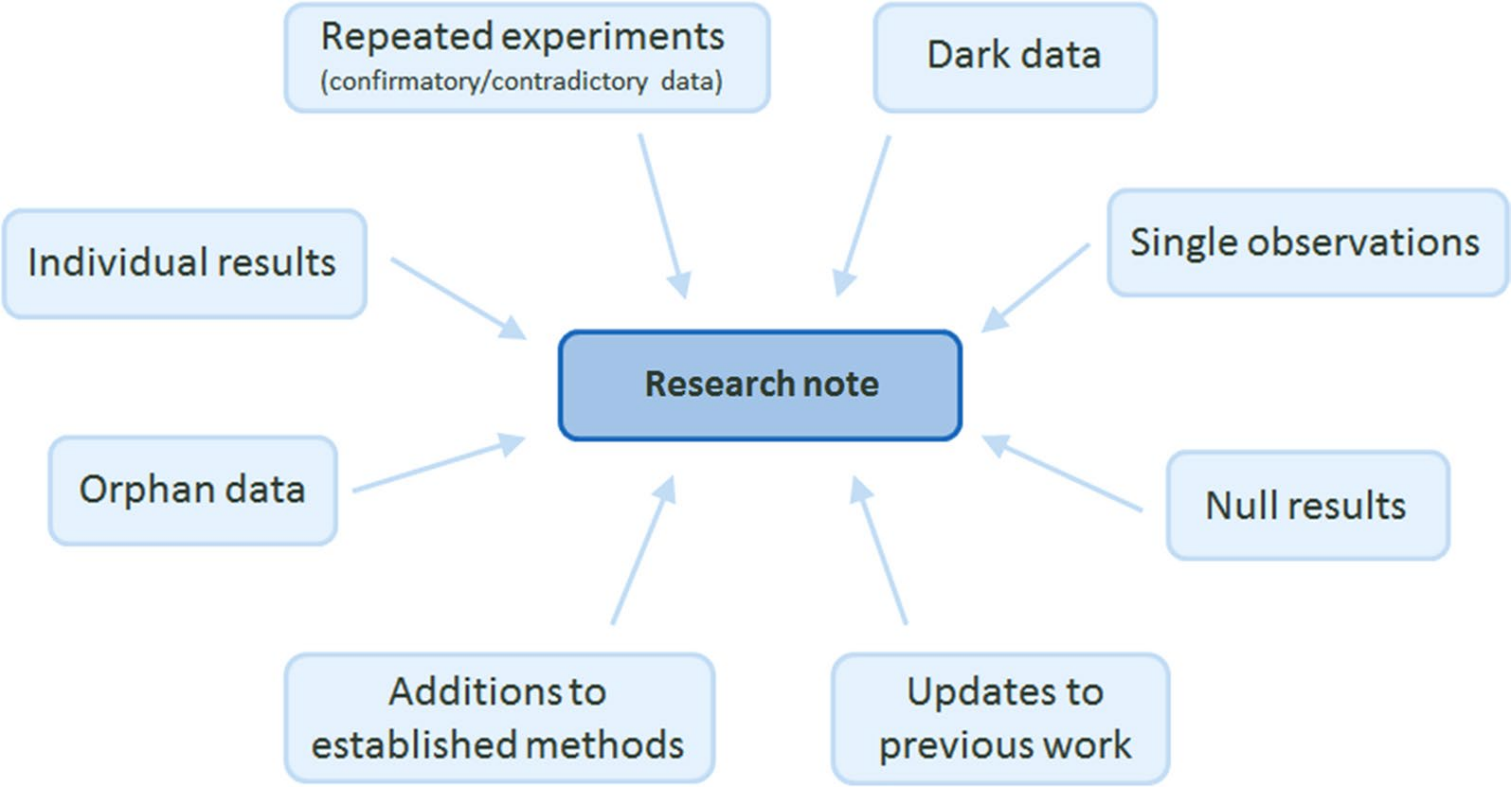
‘Research note’ is the main article type of *BMC Research Notes* and suitable for the publication of a broad range of research outputs

We have reduced the number of article types and created ‘Research note’ which will be suitable for most manuscripts submitted to the journal. ‘Research note’ is a short note article for the reporting of unconventional or brief research outputs (Fig. 1). The article type has a word limit to ensure authors focus on the essential scientific content and making it easy to digest for readers. ‘Research note’ is structured in

- Abstract
- Introduction
- Main text
- Limitations.

The abstract must be structured in ‘Objectives’ and ‘Results’. The former is particularly important and authors should clearly state the motivation, research question or origin of data.

The introduction should be brief and provide, similarly to ‘Objectives’ of the abstract, the motivation for the work presented in the manuscript (e.g., Where does the data come from? Why was the data obtained?). Authors should state if the data is a single observation or the ‘side product’ of another study. This will not negatively impact editorial assessment. We are not looking for a detailed and lengthy introduction to the topic and authors should instead cite relevant review articles. No general review of the related literature is required. Authors should cite relevant work if the manuscript extends previously published or unpublished research.

The main text section contains the body of the research note. Authors should concisely describe the data or results and provide a brief but critical discussion. Short informative headings may be used to structure the section.

The manuscript should finish with a brief description of the limitations of the work. This can be done in bullet point format and will not negatively impact publication of the manuscript. A clear description of the limitations will ensure the impact of the work is clear and of maximum benefit to the research community.

The benefits of the new article type are:

- Shorter article type with a word limit to ensure research findings are presented concisely
- Objective clearly stated in the abstract
- Focus on scientific findings without a lengthy introduction
- Clear description of limitations at the end of the research note.

### Expanded subject scope

**Fig. 2:**
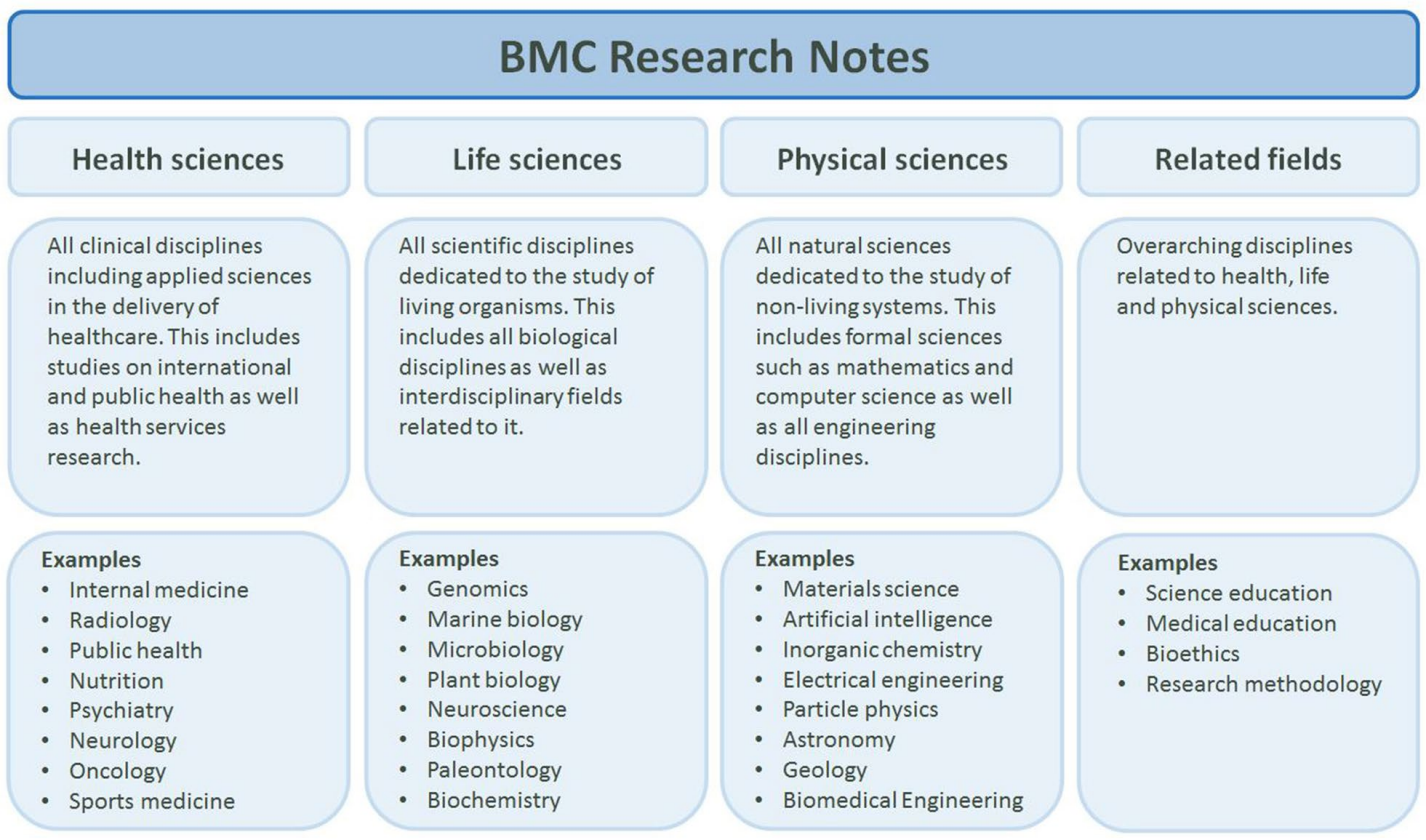
Overview of the different subject cluster. *BMC Research Notes* will consider manuscripts on health, life, and physical sciences as well as related disciplines such as bioethics. Our definition of physical sciences is very broad including all engineering disciplines as well as formal sciences such a mathematics and computer science

Dark data not only exists in biological and medical research fields but is being generated by researchers of all disciplines across the world. *BMC Research Notes* aspires to serve a broad and global research community. As a result, the journal will expand its scope to include all scientific and clinical disciplines, i.e. health sciences and medicine, life sciences, physical sciences including all engineering disciplines, computer science and mathematics as well as related disciplines such as science education, bioethics and research methodology (Fig. 2).

By expanding the scope of the journal, researchers of all scientific and clinical disciplines are able to take advantage of the innovative approach of *BMC Research Notes* to make overlooked research outputs from a broad range of subject disciplines available to the research community.

## Conclusion

Our vision for *BMC Research Notes* is to build a unique, high profile journal focusing on short research notes that will serve as a dedicated open access forum for research outputs which would otherwise remain unknown. As part of the established *BMC* series, the journal’s primary goal is to serve the global scientific and clinical research communities. Authors, reviewers, readers and those with an interest in the journal are encouraged to get in touch with the Editor and provide us with feedback and suggestions as we work to innovate and provide a service to the research community.
